# Isolated sigmoid varicose vein rupture and hemorrhage: A case report

**DOI:** 10.1097/MD.0000000000030024

**Published:** 2022-07-29

**Authors:** Weiwei Li, Jianli Wang, Hua Fu, Jinlong Liu

**Affiliations:** Department of Hepatobiliary Surgery, Affiliated Hospital of Chengde Medical University, Chengde 067000, Hebei Province, China.

**Keywords:** ectopic varices, embolization, sigmoid resection, sigmoid varicose veins, variceal bleeding

## Abstract

**Rationale:**

Ectopic varices are the collateral circulation of portal vein located anywhere in the gastrointestinal tract other than the esophageal and gastric regions. Rupture of these varices often results in life-threatening hemorrhage. Management guidelines for ectopic variceal bleeds are not yet standardized because cases are rare and treatment approaches described in the literature vary considerably.

**Patient concerns:**

A 53-year-old woman with a 20-year history of chronic hepatitis C cirrhosis came to our hospital for treatment due to intermittent black stools for 4 days. After admission, the patient developed hemorrhagic shock, with hemodynamic instability.

**Diagnosis:**

Postoperative histological examination confirmed the diagnosis of sigmoid varicose veins.

**Intervention:**

Emergency colonoscopy showed that a varicose vein mass in the sigmoid colon wall 30 cm from the anus was ruptured and bleeding. Percutaneous transhepatic inferior mesenteric venography revealed the presence of a varicose mass of sigmoid colon veins. After embolization of the sigmoid varicose veins with spring coils, angiography showed that the hemorheology of the distal varicose vein mass was slow but not completely blocked. Three days after embolization, the patient had hematochezia again. Splenectomy and sigmoid colon resection were performed immediately.

**Outcomes:**

Follow-up computed tomography showed no residual varices were observed after sigmoid colon resection.

**Lessons:**

Ectopic varices, which are rare sequelae of portal hypertension, need to be taken seriously because bleeding from these varices can be catastrophic. We report a case of isolated sigmoid variceal rupture and hemorrhage due to portal hypertension in cirrhosis. The patient experienced failure of endoscopic hemostasis and sigmoid colon venous coil embolization. She was eventually successfully brought to hemostasis by surgery.

## 1. Introduction

Ectopic varices are a rare cause of lower gastrointestinal bleeding. They are any collateral circulation of portal vein anywhere in the gastrointestinal tract outside the esophageal and gastric regions.^[1]^ These varicose veins are usually caused by portal hypertension. The most common site is the duodenum, followed by the jejunum and ileum, colon, and rectum.^[2]^ Sigmoid varicose veins are rarely reported and are usually accompanied by rectal varicose veins.^[3]^ To date, there is still no clear treatment plan, and no consensus has been reached. Choosing the appropriate treatment, including alternative treatment in case of initial treatment failure, merits considerable discussion. Here, we report a case of hemorrhagic shock in a woman with isolated sigmoid variceal rupture and hemorrhage. This patient experienced failure of endoscopic hemostasis and inferior mesenteric venous coil embolization and was eventually successfully brought to hemostasis by sigmoid resection.

## 2. Case presentation

**Figure 1.**
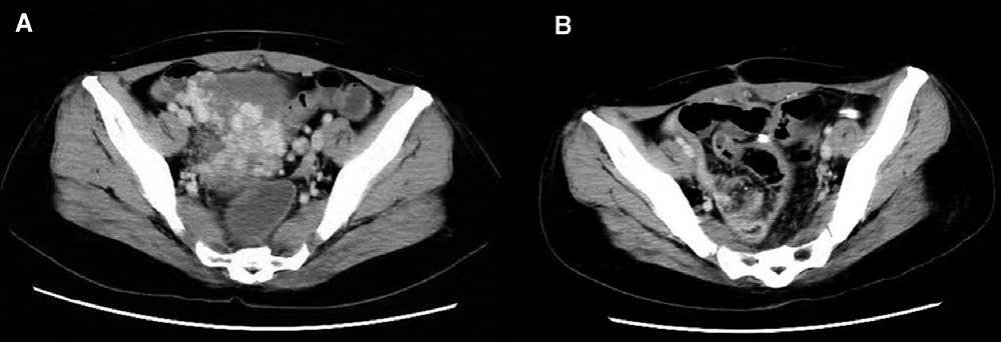
Computed tomography: (A) multiple tortuous and dilated vascular shadows in the pelvic cavity; and (B) no residual varices were observed after sigmoid colon resection.

**Figure 2.**
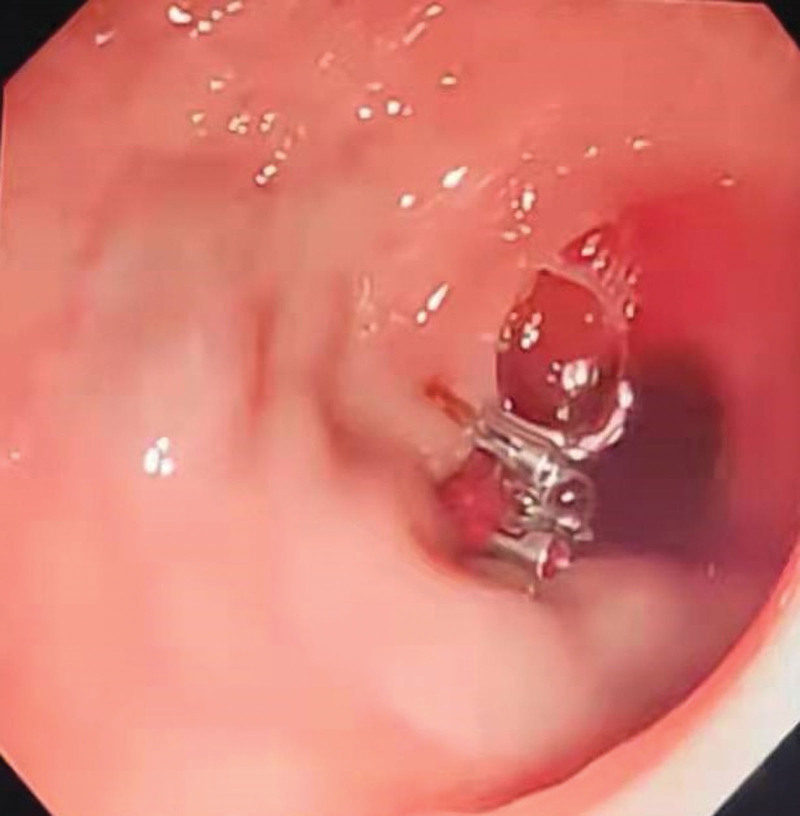
The ruptured vein mass was clipped with a titanium clip under colonoscopy.

A 53-year-old female with a 20-year history of chronic hepatitis C cirrhosis came to our hospital for treatment due to intermittent black stools for 4 days. On admission, the patient presented anemia, pallid palpebral conjunctiva, and enlarged spleen. Contrast-enhanced computed tomography showed multiple tortuous and dilated vascular shadows in the pelvic cavity, which were connected to the splenic vein (Fig. 1A). Laboratory findings were as follows: hemoglobin 91 g/L, serum albumin 23.04 g/L, total bilirubin 16.8 μmol/L, and prothrombin time 14.8 seconds. Her Child-Pugh class was B. After admission, the patient developed hemorrhagic shock again, with hemodynamic instability (HR 140 beats/min, BP 80/30 mmHg). No obvious varicose veins and bleeding were found on gastroscopy. Emergency colonoscopy found that a varicose vein mass in the sigmoid colon wall 30 cm from the anus was ruptured and bleeding. The ruptured vein mass was immediately clipted by titanium clip for hemostasis treatment (Fig. 2).

**Figure 3.**
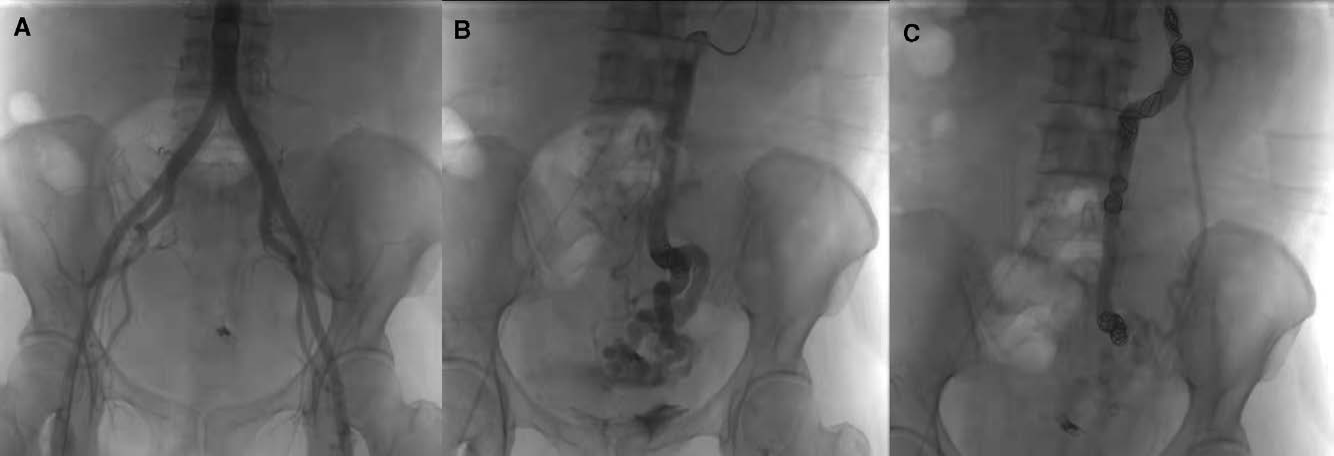
(A) Aortography showed no arteriovenous fistula; (B) percutaneous transhepatic inferior mesenteric venography revealed the presence of a varicose mass of sigmoid colon vein; and (C) slow blood flow was observed after embolization.

Due to the severity of the patient’s varicose veins, there was a risk that the clamp would fall off, and the risk of rebleeding remained high. Subsequent aortography revealed no arteriovenous fistula (Fig. 3A), and percutaneous transhepatic inferior mesenteric venography revealed the presence of a varicose mass of sigmoid colon veins (Fig. 3B). The portal vein pressure was 22 mmHg. After embolization of the sigmoid colon veins with spring coils, angiography showed that the hemorheology of the distal varicose vein mass was slow but not completely blocked (Fig. 3C).

Three days after embolization, the patient had hematochezia again. To reduce portal vein pressure and local hemostasis, splenectomy and sigmoid colon resection were performed immediately. During the operation, nodular sclerosis of liver and enlarged spleen were observed. Surrounding sigmoid colon and mesangial varicose veins were obvious, with a maximum diameter of about 1.0 cm. Postoperative histological examination confirmed the diagnosis of sigmoid varicose veins. Follow-up contrast-enhanced computed tomography showed no residual varices were observed after sigmoid colon resection (Fig. 1B). At 3-month follow-up, the patient’s hemoglobin level increased to 140 g/L and there was no further sigmoid bleeding.

## 3. Discussion

Sigmoid varicose veins are a type of ectopic varices. Although extremely rare, they should not be ignored. Ectopic variceal bleeding accounts for only 2% to 5% of gastrointestinal tract variceal bleeding. Unlike normal variceal bleeding, ectopic variceal bleeding is usually difficult to detect, rapid, and hidden. Some studies suggest that ectopic varices have a 4-fold higher increased risk of bleeding relative to esophageal varices and a mortality rate as high as 40%.^[2,4]^ In 1985, Vock et al^[5]^ reported a case of bleeding caused by ruptured sigmoid varices in liver cirrhosis that led to death within a few hours. The source of bleeding was almost invisible even microscopically. Given the improvements that have been made in relevant technology, we had many diagnostic methods available to us for this case. In this case, contrast-enhanced computed tomography imaging and colonoscopy were performed, and the diagnosis of isolated sigmoid varicose veins was confirmed by inferior mesenteric vein angiography.

A variety of treatment options are available for venous hemorrhage, including endoscopic intervention, transjugular intrahepatic portosystemic shunt (TIPS), angiographic embolization, and surgery. Because cases of sigmoid varicose veins due to portal hypertension are rare, the management of bleeding related to sigmoid varicose rupture, as in this case, is limited. There are currently no specific guidelines for treatment. The choice of treatment is usually highly time-sensitive. For the rescue treatment of early and acute hemorrhage, endoscopic therapy offers good choices, including clamps, band ligation, injection sclerotherapy, and argon plasma solidification.^[6]^ In our case, endoscopic titanium clip hemostasis was used as a preliminary measure to control acute hemorrhage from sigmoid varices. However, endoscopic treatment was found to be a good long-term solution due to the severe varicose veins, high vascular tension, and the risk of the titanium clip falling off. Further reliable treatments are needed.

TIPS and embolization are both minimally invasive endovascular interventions that may be suitable alternatives to variceal bleeding that does not respond well to endoscopic treatment. TIPS can be used to achieve hemostasis by significantly reducing portal vein pressure.^[7]^ However, some studies have shown that the efficacy of TIPS in patients with ectopic varices is not ideal.^[8]^ The 1-year estimated cumulative rate of rebleeding after TIPS in patients with ectopic varices is significantly higher than that in patients with gastroesophageal varices, reaching 23%, compared to 0% to 6% of patients with bleeding of the gastroesophageal varices. The risk of rebleeding in patients with duodenal varices was found to be as high as 50%. Given the risk of liver failure and hepatic encephalopathy after TIPS and the patient’s refusal to undergo TIPS, we chose angiographic embolization for treatment.

Variceal embolization is accomplished by occluding the vein supplying blood to the bleeding area. Ahn et al^[9]^ successfully treated intractable rectal variceal bleeding using sigmoid colon vein embolization. However, this form of treatment has only rarely been reported in isolated sigmoid varicose hemorrhage. We attempted to do this with spring coil embolization in the sigmoid colon vein via percutaneous transhepatic portal vein puncture. However, the results were not satisfactory, and the patient suffered a recurrence of bleeding 3 days later. We thought this might be due to the large diameter of the patient’s sigmoid varices, which led to the failure of embolization. We subsequently performed sigmoid resection, and there was no further bleeding after removal of the varicose vein. All this indicates that surgery can be used as an alternative treatment if embolization fails.

## 4. Conclusion

To our knowledge, this is the first reported case of isolated sigmoid variceal rupture and hemorrhage. The patient experienced failure of endoscopic hemostasis and sigmoid colon venous coil embolization. She was eventually successfully brought to hemostasis by surgery. Ectopic varices, which are rare sequelae of portal hypertension, need to be taken seriously because bleeding from these varices can be catastrophic. Further case studies are needed to establish standard treatment strategies by comparing the efficacy and safety of treatments.

## Acknowledgments

We thank LetPub (www.letpub.com) for its linguistic assistance during the preparation of this manuscript.

## Author contributions

Weiwei Li and Jinlong Liu reviewed the literature and contributed to manuscript writing and data analysis; Weiwei Li and Jinlong Liu contributed to draft conception and design; Jianli Wang, Hua Fu and Jinlong Liu were the clinicians involved in patient diagnostics, management, therapy, and surveillance; all authors have read and approved the final manuscript.
